# Health related quality of life of children with rheumatic heart diseases: reliability of the Brazilian version of the pediatric quality of life inventory™ cardiac module scale

**DOI:** 10.1186/1477-7525-11-198

**Published:** 2013-11-15

**Authors:** Anabela do Nascimento Moraes, Maria Teresa Ramos Ascensão Terreri, Maria Odete Esteves Hilário, Cláudio Arnaldo Len

**Affiliations:** 1 Pediatric Service, Medical School of the Federal University of Pará, Bettina Ferro de Souza University Hospital (Universidade Federal do Pará - FAMED/UFPA/HUBFS), Pará, Brazil; 2 Rheumatology Unit, Department of Pediatrics, Federal University of São Paulo/São Paulo School of Medicine (Universidade Federal de São Paulo/Escola Paulista de Medicina – UNIFESP/EPM), São Paulo, Brazil

**Keywords:** Quality of life, Heart diseases, Rheumatic fever, Validation studies, Translation, Questionnaires

## Abstract

**Background:**

This study aimed to translate the ‘Pediatric Quality of Life Inventory™ (PedsQL™ 3.0) Cardiac Module’ into Portuguese, adapt it to Brazilian culture, and assess its psychometric properties (validity and reproducibility), and to calculate health-related quality of life scores on the PedsQL 4.0 and PedsQL™ 3.0 Cardiac Module Scales for a group of patients 5 to 18 years old with rheumatic heart disease.

**Methods:**

The methods suggested by the authors of the original version of the questionnaire included 1) translation by an expert panel; 2) translation back into English and revision by the authors of the original version; 3) pilot study with seven children and parents in each of three age ranges (5 to 7, 8 to 12, and 13 to 18 years old); and 4) assessment of the measurement properties. In this stage, the PedsQL™ 3.0 Cardiac Module and the PedsQL 4.0 Generic Scale were applied to a sample comprising 109 children and adolescents with rheumatic heart disease and their parents or caregivers. The version for parents or caregivers was administered separately on the same day.

**Results:**

The values of Cronbach’s alpha for all scales assessed in the questionnaire (heart problems and treatment [symptoms], problems with perceived physical appearance, treatment anxiety, cognitive problems, and communication problems) varied from 0.6 to 0.8, indicating good internal consistency. Correlation was found between the scores for the Cardiac Module and the Generic Scale (0.36-0.86), demonstrating convergent validity (Spearman’s correlation coefficient, p < 0.01). The symptoms, problems with perceived physical appearance, and cognitive and communication problem domains were able to distinguish between groups of patients with mild and moderate/severe heart disease (Student’s t-test, p < 0.05). The intraclass correlation of the interobserver reproducibility was adequate (0.76 to 0.94 among the patients [children/adolescents] and 0.76 to 0.84 among their caregivers). The correlation between the patients’ scores and their parents’ scores varied from 0.50 to 0.86 (Pearson’s correlation coefficient, p < 0.01).

**Conclusions:**

The Brazilian version of the PedsQL™ 3.0 Cardiac Module was shown to be reliable. The application of this questionnaire in practice will be very useful for all professionals charged with the care of children and adolescents with heart diseases.

## Background

Rheumatic fever is an important cause of acquired heart disease among children, adolescents, and young adults in developing countries and is therefore a relevant public health problem. Its high morbidity and mortality indexes and significant social and economic costs must be emphasized. Every year, approximately 15 million people are affected, 200,000 die, and 100,000 become disabled [1-3].

Congenital or acquired cardiovascular diseases exert negative impacts on the health-related quality of life (HRQOL) of patients and their relatives [4-8].

Advances in the clinical and surgical treatment of congenital and acquired heart diseases have contributed to a significant reduction in mortality rates in recent decades [9,10], thus increasing patients’ life expectancies. However, in spite of the increased survival associated with advances in medical and surgical care, problems such as organ dysfunction [11,12], psychosocial disorders [4], and effects on neurological development [13] might still occur, limiting patients’ cognitive development and their productivity in adult life [14]. Therefore, one of the aims pursued by health care providers and promoters is to increase the scores on the physical, emotional, social, and school-related dimensions [15].

The various dimensions of the HRQOL on patients with heart disease might be assessed using generic or specific questionnaires. The *Pediatric Quality of Life Inventory™3.0 Cardiac Module*^16^ (PedsQL™ 3.0 Cardiac Module) is a specific instrument that can be easily and quickly applied to measure HRQOL in children and adolescents with heart disease. It is validated for use in clinical practice, where it facilitates the identification of risk, health status, and the results of treatment in pediatric populations with heart disease [16,17].

There are no specific instruments available to measure HRQOL for children with heart disease in Brazil. Therefore, the aims of the present study are as follows: to translate and validate the PedsQL™ 3.0 Cardiac Module for the Brazilian Portuguese language and culture and establish the HRQOL scores on the PedsQL 4.0 and PedsQL™ 3.0 Cardiac Modules in a group of children with rheumatic heart disease at a reference outpatient clinic.

## Methods

The present study was approved by the local ethics and research committee of the Federal University of São Paulo. All the patients and/or their parents or caregivers agreed to participate and signed an informed consent form.

The following guidelines suggested by the authors of the original version of the PedsQL™ 3.0 Cardiac Module [18] were followed in the process of translation and validation:

Stage 1. The original English version of the PedsQL™ 3.0 Cardiac Module was independently translated into Portuguese by three investigators from the field of health (A.N.M., C.A.L., M.O.E.H.), followed by a joint discussion to combine the three independent versions into a single version. In this stage, the efforts of the investigators were devoted to achieving linguistic and conceptual equivalence.

Stage 2. The initial consensus Portuguese version was translated back into English by two bilingual translators. The back translation was sent to the PedsQL project staff, chaired by Dr. James Varni in San Diego, for appraisal and revision. In a second discussion, the Brazilian investigators accepted and included the changes and amendments suggested by the San Diego team in the Portuguese version.

Stage 3. Pilot study. The version of the PedsQL™ 3.0 Cardiac Module that was translated and adapted to the Brazilian Portuguese language and culture was applied to 21 patients with rheumatic heart disease at an outpatient setting using the cognitive method prescribed by the MAPI Research Institute.

Seven children in each age range (5 to 7, 8 to 12, and 13 to 18 years old) and their parents were interviewed, for a total of 42 individuals, to assess their responses to the questions and identify possible mistakes and difficulties resulting from the translation. The questionnaires were administered before medical appointments in a quiet environment (meeting room). Application of the questionnaire lasted an average of 5 to 10 minutes for both children and adults.

The cross-cultural adaptation of the questionnaire was performed during this stage to achieve semantic equivalence (equivalence between words), idiomatic equivalence (equivalent expressions or items needing substitution), and experimental equivalence (words and situations appropriate to the Brazilian cultural context). The first version was thus corrected for later use in the assessment of the instrument’s psychometric properties.

Stage 4. This stage consisted of a field study in which the questionnaires were administered to a larger number of patients stratified by age range and their parents.

### Inclusion criteria

#### Patients

•Age: 5 to 18 years old

•Diagnosed with rheumatic fever according to the modified Jones criteria [19].

•Diagnosed with chronic rheumatic heart disease [20,21].

•Without any other physical and/or mental comorbidity.

•Escorted by his or her father, mother, or other caregiver to the medical appointment

Demographic and socioeconomic status data of the patients and their parents or caregivers were collected at this stage in a protocol based on the criteria formulated by the Brazilian Association of Market Research Companies: low socioeconomic class family (E, D, C), middle class family (B) and high class family (A) [22].

The PedsQL™ 3.0 Cardiac Module presents a list of items to the children/parents and asks them to answer how problematic each item has been to the children in the past month. Five answer options are listed in the versions for children 8 to 18 years old and their parents (0 = it is never a problem; 1 = it is almost never a problem; 2 = it is sometimes a problem; 3 = it is often a problem; and 4 = it is almost always a problem). The answers in the version for children 5 to 7 years old was simplified to three options (0 = not at all; 2 = sometimes; and 4 = a lot) on a visual analog scale that exhibited a happy, neutral, and sad face, respectively.

The items were reverse scored and linearly transformed to a 0–100 scale (0 = 100; 1 = 75; 2 = 50; 3 = 25; and 4 = 0) so that higher scores indicated a better HRQOL. The mean score was computed as the sum of all the items over the number of items answered (taking into account the missing data).

To investigate the interobserver reproducibility of the Brazilian version of the PedsQL™ 3.0 Cardiac Module, the questionnaire was applied to a group of patients (n = 42) and their caregivers (n = 42) separately by two independent investigators on the same afternoon. To investigate intraobserver agreement, the instrument was applied twice to the 42 patients and their parents by the same investigator after a 7- to 10-day interval.

To evaluate the construct validity and reliability, the following parameters were assessed in all the patients:

•Degree of cardiac involvement in the acute stage (so-called carditis): 0 = no carditis; 1 = mild carditis; 2 = moderate carditis; and 3 = severe carditis. Severity of heart disease was rated by an assistant clinician pediatric cardiologist [23,24].

•Presence and degree of cardiac involvement in the chronic stage (so-called rheumatic heart disease), according to the echocardiographic standard established by Feigenbaum [20] and Assef [21]: 0 = minimal; 1 = mild; 2 = moderate; 3 = severe; and 4 = surgical treatment of valve disease secondary to oral foci of infection.

### Statistical study

First, the sociodemographic and clinical variables were subjected to descriptive analysis. The numerical variables were expressed as the means and standard deviations (SD), and the categorical variables were expressed as absolute and relative frequencies. Next, the homogeneity of the distribution of the categorical variables was investigated by means of the chi square test. In the case of small samples where the chi square test was compromised (more than 20% expected cell count of less than five), the Fisher exact test was used.

The psychometric tests investigated features related to the instrument’s reliability and validity.

The internal consistency of the PedsQL™ 3.0 Cardiac Module Scale and Subscales was assessed by means of Cronbach’s alpha. Scales with reliability ≥ 0.70 are recommended to compare groups of patients, whereas 0.90 is recommended for analyzing the patients’ individual scores. In practice, values ≥ 0.50 might be taken into consideration [25]. To assess the correlation of each item with its corresponding subscale, Spearman’s correlation coefficient was calculated; results ≤ 0.30 were classified as exhibiting low correlation, > 0.30 – 0.49 as moderate, and > 0.50 as high [26].

The reproducibility was investigated by calculating the intraclass correlation coefficient (ICC) relative to the scores from the six subscales. For that purpose, 95% confidence intervals (95% CI) were calculated. The ICC was determined by means of analysis of variance for random effect, and the results were classified as follows: ≤ 0.40 = weak correlation, 0.41-0.60 = moderate correlation, 0.61-0.80 = good correlation, and 0.81-1.00 = excellent correlation [27,28].

The construct validity was assessed by means of discriminant and convergent validity.

Discriminant validity was investigated by comparing the scores of different groups of patients according to the severity of disease (mild versus moderate/severe rheumatic heart disease). According to the developed hypothesis, the patients with moderate/severe heart disease would exhibit lower scores on the PedsQL™ 3.0 Cardiac Module domains compared with patients with mild rheumatic heart disease. For that purpose, Student’s t-test was used (independent samples by age).

Convergent validity was assessed by means of the intercorrelation of the scores on the Cardiac Module with the relevant scores on the PedsQL™ 4.0 Generic Scale. On these grounds, the following hypothesis was elaborated: heart problems and treatment (symptoms) might be correlated with perceived physical appearance; problems with perceived physical appearance might be correlated with psychosocial functioning; cognitive problems might be correlated with school functioning; and treatment anxiety might be correlated with psychosocial functioning. These correlations were tested using Spearman’s correlation coefficient.

The correlation between the patients’ and the corresponding caregivers’ scores was investigated using Pearson’s and Spearman’s correlation tests. In Pearson’s correlation (r), r ≥ 0.30 was considered moderate, and r ≥ 0.50 was considered high.

In all analyses, a probability ≤ 0.05 of alpha error was considered statistically significant.

Statistical analysis was performed using the SPSS software for Windows (version 12.0) and Microsoft Excel.

## Results

### Sample characteristics

The study encompassed all participants that met the inclusion criteria, for a total sample comprising 109 children and adolescents and their parents.

The distribution per age range was homogeneous, with 29 patients (6.6%) who were 5 to 7 years old and 40 patients (36.7%) who were 8 to 12 and 13 to 18 years old, with a slight predominance of females (52.3%). Approximately 67.9% (N = 74) of the sample had mild heart disease, 22.0% (N = 24) of the sample had moderate heart disease and 10,0% (N = 11) of the sample had severe heart disease; the severe form with valve disease prevailed among the adolescents.

The questionnaire was preferentially applied to mothers (89.0%), and most families belonged to the lower economic classes (93.6% of low socioeconomic class family). Concerning the caregivers’ educational levels, 57.8% had attended secondary school, albeit incompletely, and 19.3% had incomplete elementary education or less (illiterate); however, all of the patients attended school.

Despite the low educational levels, the self-reporting procedure was selected for application of the questionnaires, and orientation was given to alleviate potential doubts. Verbal application of the questionnaire was needed for six patients (7.5%) who were 8 to 18 years old and for 12 parents or caregivers (11.0%).

Concerning the clinical and/or laboratory activity of disease, seven cases (6.4%) exhibited acute carditis, three (2.7%) exhibited mild carditis, two (1.8%) exhibited moderate carditis, and two (1.8%) had severe activity.

### Descriptive statistics

The PedsQL 4.0 and PedsQL™ 3.0 Cardiac Module scores of the patients and their parents are described in Table 1. The psychosocial health dimension exhibited lower scores, both in the children’s and the parents’ perception, and the school functioning domain exhibited the lowest score. The same tendency was found in the assessment of HRQOL using the specific scale: the non-physical domains, which include perceived physical appearance, treatment anxiety, and cognitive and communication problems, exhibited lower scores compared with physical functioning (heart problems and treatment/symptoms) in both the children’s and the parents’ assessments.

**Table 1:** **Scale descriptives for the pediatric quality of life inventory**™ **generic core scale and pediatric quality of life inventory**™ **3.0 cardiac module child self-report and parent proxy-report**

**Scale**	**No. items**	**Self-report**			**Proxy-report**		
		**Mean**	**SD**	**N**	**Mean**	**SD**	**N**
*Generic core scale*							
*Total scale score*	*23*	*72.9*	*13.7*	*109*	*69.6*	*15.5*	*109*
Physical	8	82.6	19.2	109	80.1	21.3	109
Emotional	5	70.1	15.3	109	64.7	15.9	109
Social	5	78.2	17.1	109	74.9	19.7	109
School	5	55.0	23.0	109	52.2	27.2	109
Psychosocial	15	67.8	13.5	109	63.9	15.5	109
*Cardiac module*							
*Total scale score*	27 (25)*	*72.8*	*15.0*	*109*	*68.9*	*16.0*	*109*
Heart problems and treatment	7	78.5	19.9	109	77.1	22.7	109
Problems with treatment	5 (3)*	82.0	20.5	109	80.8	19.7	109
Physical appearance	3	65.0	30.3	109	66.9	30.9	109
Treatment anxiety	4	74.5	29.6	109	57.2	30.9	109
Cognitive problems	5	66.1	25.3	109	63.7	27.4	109
Communication problems	3	61.4	15.0	109	57.9	32.8	109

Psychosocial: sum of the emotional, social, and school scales. (25)* and (3)*: Number of items for children 5 to 7 years old. Higher scores on the PedsQL 4.0 Generic Scale and the PedsQL™ 3.0 Cardiac Module indicate fewer difficulties/limitations and better HRQOL.

### Internal consistency reliability

Assessment of the internal consistency of the items that compose each PedsQL™ 3.0 Cardiac Module Scale is described in Table 2. The values for Cronbach’s alpha were greater than 0.8; however, they were lower than 0.9 in the items relative to heart problems and treatment (symptoms) in the caregivers’ assessment and treatment anxiety in both the children’s and parents’ assessments, which indicates that the questionnaire has good internal consistency.

**Table 2:** **Internal consistency reliability for the pediatric quality of life inventory**™ **3.0 cardiac module scale versions for child self-report and parent proxy-report**

**Domains**	**Cronbach’s alpha**			
	**Self-report**	**N**	**Proxy-report**	**N**
Heart problems and treatment	0.76	109	0.82	109
Problems with treatment	0.52* 0.70**	109	0.58* 0.08**	109
Physical appearance	0.72	109	0.75	109
Treatment anxiety	0.85	109	0.89	109
Cognitive problems	0.66	109	0.73	109
Communication problems	0.75	109	0.70	109
*Total sample*	0.84* 0.77**	*109*	0.85* 0.80**	*109*

p < 0.01.

*Cronbach’s alpha for a group of 80 patients 8 to 18 years old.

**Cronbach’s alpha for a group of 29 patients 5 to 7 years old.

Acceptable values for the alpha coefficient in heart problems, problems with perceived physical appearance, and problems with communication domains in the children’s self-assessment, and in problems with physical appearance, cognitive problems, and communication problems in the parents’ assessment. Conversely, cognitive problems according to the children’s perception and treatment problems according to both parents and the children 5 to 18 years old exhibited poor coefficients.

### Test-retest reliability

The values obtained in the analysis of reproducibility of the PedsQL™ 3.0 Cardiac Module Subscales are described in Table 3. The patients in the three age ranges and their parents were grouped together, for a total of 84 individuals. Based on the patients’ self-reports and excluding the cognitive problems subscale, all the scales exhibited satisfactory correlation, with ICC > 0.8. In the case of the caregivers, the total scale and each individual domain exhibited good correlation.

**Table 3:** **Intraclass correlations of the pediatric quality of life inventory™ 3.0 cardiac module scale for child self-report (42 subjects) and parent proxy-report (42 subjects)**

**Domains**	**Intraclass correlation (95% CI)**			
	**Self-report**		**Proxy-report**	
	**Interobs**	**Intraobs**	**Interobs**	**Intraobs**
Heart problems and treatment	0.94	0.93	0.84	0.84
Problems with treatment	0.94	0.90	0.77	0.83
Physical appearance	0.91	0.91	0.81	0.78
Treatment anxiety	0.93	0.90	0.77	0.84
Cognitive problems	0.76	0.78	0.76	0.82
Communication problems	0.87	0.90	0.78	0.88
*Total*	*0.93*	*0.91*	*0.79*	*0.92*

CI = confidence interval, Interobs = Interobserver, Intraobs = Intraobserver.

### Construct validity

To assess the discriminant validity of the PedsQL™ 3.0 Cardiac Module, the scores for two different groups of patients categorized by the severity of disease (mild versus moderate/severe) were compared. The average and total scores for the physical domain (heart problems and treatment/symptoms) exhibited significant correlation with moderate/severe heart disease, as expected, in both the patients’ self-assessment and the parents’ proxy assessment (Table 4).

**Table 4:** **Analysis of the average scores for the pediatric quality of life inventory**™ **3.0 cardiac module scale domains by disease severity**

**Domains**	**Mild RHD**	**Moderate/severe RHD**	**t (p-value)**
	**Mean (SD)**	**Mean (SD)**	
*Cardiac module*			
*Child self-report*			
*Total sample*	*75.2 (12.8)*	*67.6 (17.9)*	*2.24 (p = 0.0295)*
Heart problems and treatment	82.3 (17.7)	70.6 (22.1)	2.74 (p = 0.0083)
Problems with treatment	82.3 (22.0)	81.3 (17.0)	0.23 (p = 0.8149)
Physical appearance	67.5 (28.2)	59.8 (34.1)	1.24 (p = 0.2174)
Treatment anxiety	77.3 (28.4)	68.8 (31.5)	1.41 (p = 0.1605)
Cognitive problems	68.2 (24.1)	61.6 (27.7)	1.27 (p = 0.2054)
Communication problems	64.1 (31.4)	55.7 (37.3)	1.22 (p = 0.2248)
*Parent proxy-report*			
*Total sample*	*73.4 (13.8)*	*59.5 (16.5)*	*4.62 (p < 0.0001)*
Heart problems and treatment	82.7 (20.1)	65.3 (23.5)	3.77 (p = 0.0004)
Problems with treatment	81.8 (20.6)	78.5 (17.6)	0.83 (p = 0.4062)
Physical appearance	72.5 (29.3)	55.0 (31.3)	2.85 (p = 0.0053)
Treatment anxiety	60.9 (34.3)	49.5 (33.3)	1.64 (p = 0.1042)
Cognitive problems	69.5 (24.4)	51.4 (29.5)	3.36 (p = 0.0011)
Communication problems	62.8 (30.6)	47.6 (35.3)	2.29 (p = 0.0237)

RHD = rheumatic heart disease, SD = standard deviation.

The scores for the non-physical domains of the patients did not correlate with the severity of disease. The parents’ scores relative to the non-physical domains correlated significantly with the severity of disease, except for treatment anxiety (Table 4).

The convergent validity (Table 5) of the PedsQL™ 3.0 Cardiac Module was assessed by means of analysis of the intercorrelation between the scores in the Cardiac Module Subscales and the scores for physical, psychosocial, and school functioning in the PedsQL™ 4.0 Generic Scale. According to the formulated hypothesis, the scores for subscale heart problems and treatment (symptoms) exhibited strong and significant correlation with the physical scales, r = 0.85 for the patients and r = 0.86 for the parents (p < 0.01). The perceived physical appearance domain exhibited moderate but significant correlation with psychosocial functioning, r = 0.36 for the patients and r = 044 for the parents (p < 0.01). The scores for the subscale cognitive problems exhibited significant correlation with school functioning, r = 0.72 and r = 0.77 for children and parents, respectively (p < 0.01). Correlation was found between treatment anxiety and psychosocial functioning (r = 0.38 for the children and r = 00.39 for the parents, p < 0.01).

**Table 5:** **Intercorrelations of subscales of to the pediatric quality of life inventory™ 4.0 generic core scales and cardiac module assessed with spearman**’**s correlation coefficient**

		**Cardiac module**					
	**Total sample**	**Symptoms**	**Treatment problems**	**Physical appearance**	**Treatment anxiety**	**Cognitive**	**Communication**
Generic scale							
Self-report							
*Total sample*	0.69**	0.72**	*-0.06*	0.42**	0.35**	0.44**	0.38**
Physical	0.57**	0.85**	-0.20*	0.46**	0.21*	0.31**	0.30**
Emotional	0.36**	0.26**	-0.002	0.28**	0.41**	0.03	0.19*
Social	0.48**	0.57**	0.051	0.33**	0.25**	0.22*	0.22*
School	0.61**	0.46**	-0.006	0.20*	0.24*	0.72*	0.44*
Psychosocial	0.67**	0.56**	-0.002	0.36**	0.38**	0.51**	0.39**
Proxy-report							
*Total sample*	0.72**	0.77**	*-0.11*	0.52**	0.30**	0.57**	0.26**
Physical	0.54**	0.86**	-0.22*	0.49**	0.10	0.33**	0.14
Emotional	0.45**	0.35**	0.03	0.29**	0.53**	0.17	0.14
Social	0.43**	0.52**	-0.16	0.44**	0.21*	0.25**	0.15
School	0.63**	0.43**	0.06	0.26**	0.20*	0.77**	0.26**
Psychosocial	0.71**	0.59**	0.002	0.44**	0.39**	0.62**	0.29**

Psychosocial: sum of the emotional, social, and school scales. *p < 0.05, **p < 0.01 level (2-tailed).

Child self-report (N = 109), Parent proxy-report (N = 109).

### Parent–child agreement

The perception of caregivers relative to the patients’ QOL is shown in Table 6. There was a strong association between the parents’ and patients’ perceptions relative to all aspects and in the total QOL. Whenever the children indicated better QOL, their parents followed that tendency.

**Table 6:** **Agreement between child self**-**report and parent proxy**-**report pediatric quality of life inventory**™ **3.0 cardiac module scale**

**Domains**	**Coefficient of pearson’s correlation between child self-report and parent proxy-report**		
	**r**	**N**	**p**
Heart problems and treatment	0.86	109	< 0.01
Problems with treatment	0.62	109	< 0.01
Physical appearance	0.71	109	< 0.01
Treatment anxiety	0.50	109	< 0.01
Cognitive problems	0.68	109	< 0.01
Communication problems	0.60	109	< 0.01
*Total*	*0.66*	*109*	*< 0.01*

## Discussion

The present study describes the psychometric properties of the Brazilian version of the PedsQL™ 3.0 Cardiac Module. The results attest to the reliability, reproducibility, and validity of that version for the assessment of the HRQOL of children 5 to 18 years old and of their parents or caregivers within the context of pediatric cardiology.

The contextualization of the problems (domains) was understood by both the patients and their parents. Despite the low degree of schooling of some patients and their caregivers, the self-reporting method was selected, and assistance was given to patients or parents in case they had doubts, as in the original study [29].

Among the universe of heart diseases that affect children, a homogeneous group of patients with rheumatic heart disease was chosen for the validation study of the questionnaire because it is a disabling disease [2] with negative impacts on the QOL of patients and their relatives [5-8] due to the chronic valve disease, whose tertiary prophylaxis represents 35% of the surgical procedures for cardiovascular diseases in Brazil [30].

The patients’ scores on the Generic Scale were lower compared with a small sample of healthy Brazilian children [31] and the normative US population [16] relative to the physical and psychosocial dimensions perceived by both parents and children. Although the PedsQL 4.0 was not applied to local healthy control groups, the previously mentioned differences suggest that the PedsQL 4.0 Generic Scale can distinguish between the HRQOL of healthy children and those with heart disease, as in the original version [16].

Comparison of the Generic Scale scores of the various groups of children with heart disease showed that the physical functioning of the present sample was similar to that observed by Uzark et al. [16] and slightly better than the sample described by Berkes et al. [17]. This observation is most likely related to the large number of children with mild heart disease who did not need specific medication. However, the psychosocial functioning scores were lower compared with the scores of children from the US [16] and Hungary [17].

The same tendency was found in the HRQOL scores from the specific scale (PedsQL™ 3.0 Cardiac Module) compared with children from the US^16^ and Hungary [17].

Just as in the case of the Generic Scale, the average score from the physical domain might be related to the fact that 67.9% of the sample included patients with mild disease. The physical functioning of children with mild heart disease does not significantly differ from the scores of healthy children, as shown by Uzark et al. [5,16].

Although heart disease exerts direct influence on physical health from the medical point of view, most studies on HRQOL also report low scores in the psychosocial dimensions [5,6,16,17,32], as were found in the present study. These findings might point to the “new hidden morbidity” [33], meaning problems that affect psychosocial health, whose identification is crucial for the promotion of integrated healthcare for children.

High scores in the social functioning dimension possibly indicate successful integration of children and adolescents with heart disease with their peers [34]. Low scores in the emotional dimension reflect the suffering of children from their chronic condition [8].

Although all the patients in the present study attend school, the school functioning dimension was the most affected by disease. However, it should be noted that the low scores in the school functioning dimension might also be the result of the educational conditions of the Brazilian population.

The Brazilian version of the PedsQL™ 3.0 Cardiac Module was shown to be reliable, similar to the Hungarian version [17]. The reliability of the internal consistency between the parents’ perceptions and the children 5 to 18 years old was higher than the standard minimum value of the alpha coefficient for group comparisons. The total internal consistency of the PedsQL™ 3.0 Cardiac Module was considered good based on Cronbach’s alphas of 0.84 and 0.85 relative to the patients and the parents, respectively.

The cognitive problems subscale as assessed by the patients from all three age ranges, the treatment problems subscale as assessed by patients who were 8 to 18 years old, and the treatment problems subscale as assessed by the parents of patients from all three age ranges exhibited poor reliability and internal consistency. The small size of the sample most likely interfered with the precision of the results [5,16]. In addition, the Cronbach’s alpha values might be influenced by the educational level of the sample [35]. Cronbach’s alpha was higher than 0.70 in the remainder of the scales, which means that each separate subscale might be used to measure specific domains of the HRQOL of patients with heart disease.

Analysis of the item-total correlation using Spearman’s correlation coefficient showed that the instrument’s homogeneity is satisfactory. The value of the coefficient correlation was lower than 0.30 in only two cases: the physical domain (heart problems and treatment) and problems with treatment.

In the physical domain, the item “My lips turn blue when I run” (r = 0.23, patients) exhibited low correlation because cyanosis is not a component of the investigated disease. In the problems with treatment, the item “My heart medication makes me feel sick” (r = 0.26, children, r = 0.09, parents of children 5 to 7 years old) exhibited low correlation because 67.9% of the sample had mild disease and did not use specific medication but only performed secondary prophylaxis.

The intraclass correlation used to assess the reproducibility of all scales and the total score was good; its value varied from 0.76 and 0.94. This measurement property is important to establish whether an instrument is reproducible over time, i.e., whether the scores are similar in the same individual at different moments, provided his or her clinical state has not changed.

The interval between any two measurements must be long enough to reduce the memory artifacts and short enough to reduce possible systemic alterations. Even in an arbitrary manner, intervals of 7 to 14 days are recommended [27,28].

Other studies have used analysis of discriminant validity as a model to distinguish between groups known to be different [16,17,36]. The results of the present study strengthen the hypothesis that children with more severe heart disease exhibit lower scores on the PedsQL™ 3.0 Cardiac Module compared with children with milder disease. Therefore, greater severity of disease was associated with poorer QOL and greater limitations and difficulties.

The symptoms scale was able to distinguish significantly between both groups of patients in the patients’ self-assessment and the parents’ proxy assessment; the average score for this item was lower (65.3) in the patients with moderate/severe disease, according to their parents’ judgment. Parents tend to express lower expectations, underestimate physical abilities, and overestimate the impact of physical functioning on the psychosocial wellbeing of their children, as shown by Casey et al. [37], after comparing the results of ergometric tests with the parents’ estimates of their children’s tolerance to exercise.

The patients with moderate and severe heart disease exhibited lower HRQOL scores on the PedsQL™ 3.0 Cardiac Module as a function of the limitations imposed by the disease on everyday activities. In addition, the non-physical domains were also affected, such as problems with physical appearance and cognitive and communication problems, which might be due to neuropsychological deficits associated with the disease itself or its treatment [8,37,38].

The correlations between the Cardiac Module Scales and the Generic Scale were significant, which agrees with the formulated hypothesis, and the convergent validity of the PedsQL™ 3.0 Cardiac Module was demonstrated, as the original^16^ study and other studies [17,36] have also shown.

Despite its statistical significance, the correlation among the patients between problems with perceived physical appearance and the emotional dimension was low, which might indicate the presence of hidden anxiety or distress. The feeling of being different from peers or of experiencing discrimination by peers due to the presence of scars on the chest might contribute to such findings.

Analysis of the correlation between the patients’ and caregivers’ scores revealed strong and significant agreement in all the Cardiac Module domains, which agrees with the reports in the literature [16,17,39,40]. The highest correlation corresponded to the physical domain (r = 0.86), and the lowest correlation corresponded to the non-physical domains, varying from r = 0.50 (treatment anxiety) to r = 0.71 (problems with perceived physical appearance).

The results show that the parents’ perceptions relative to the HRQOL of children with heart disease as assessed by the PedsQL™ 3.0 Cardiac Module are reliable. However, the assessments of non-physical domains, such as treatment anxiety, communication problems, cognitive problems, and problems with perceived physical appearance, are difficult to measure due to their subjective nature. This fact strengthens the need to apply specific questionnaires on QOL to children and adolescents, rather than to their parents or caregivers alone.

The limitations of the present study must be emphasized. The inclusion criteria resulted in a small sample size because the homogeneity of the sample was prioritized by selecting one single type of heart disease. The results would have been richer and improved the reliability of some domains, such as problems with treatment, if other types of heart disease, including congenital types, would have been included.

The lack of validation studies for instruments that assess the HRQOL of children and adolescents with heart disease in Brazil hindered comparison of the results. In addition, only the Hungarian version of the PedsQL™ 3.0 Cardiac Module has been validated.

Finally, the Brazilian version of the PedsQL™ 3.0 Cardiac Module exhibited adequate reliability, reproducibility, and construct validity. The results of the present study suggest that the PedsQL™ 3.0 Cardiac Module might be used as a parameter to measure the impact of heart disease on the QOL of children and adolescents.

The scores on the physical and non-physical domains confirm the need to subject children and adolescents with rheumatic heart disease to global assessment of their HRQOL. In cases where the scores from the physical domain do not agree with the severity of disease, communication to elucidate misconceptions and soothe fears, ergometric tests to provide reassurance, and prescriptions of exercise or rehabilitation might improve psychosocial QOL.

Identification of problems in the domains of perceived physical appearance, treatment anxiety, and communication problems involves referral to psychosocial treatment programs and the need to enhance participation in social activities, such as camps.

Early identification of low scores in the cognitive domain, including attention problems, indicates the need for more thorough assessment, including neuropsychological evaluation.

## Conclusions

According to the results of the present study:

The Brazilian version of the PedsQL™ 3.0 Cardiac Module is reliable, valid, and reproducible.

The sample of Brazilian children and adolescents with rheumatic heart disease exhibited low scores in all HRQOL domains.

The children and adolescents with moderate and severe heart disease exhibited lower scores in all HRQOL domains compared with patients with mild disease, as expected.

## Competing interests

The authors have no relevant financial relationships to disclose in the present article.

## Authors’ contributions

ANM conceived the study, collected the data, conducted the analysis, drafted and revised the manuscript. MTRAT, MOEH and CAL contributed to the design of the study; interpretation of the data and analyses; and revised the article for important intellectual content. All authors gave final approval of the version to the published.
